# Propensity score adjusted comparison of three-factor versus four-factor prothrombin complex concentrate for emergent warfarin reversal: a retrospective cohort study

**DOI:** 10.1186/s12873-020-00386-z

**Published:** 2020-11-26

**Authors:** David J. Margraf, Scott Seaburg, Gregory J. Beilman, Julian Wolfson, Jonathan C. Gipson, Scott A. Chapman

**Affiliations:** 1 Department of Experimental and Clinical Pharmacology, College of Pharmacy, University of Minnesota, 7-115E Weaver-Densford Hall, 308 Harvard St. SE, Minneapolis, MN 55455 USA; 2 Department of Pharmacy Services, North Memorial Health Hospital, Robbinsdale, MN USA; 3 Division of Critical Care & Acute Care Surgery, Department of Surgery, University of Minnesota, Minneapolis, MN USA; 4 Division of Biostatistics, School of Public Health, University of Minnesota, Minneapolis, MN USA; 5 Trauma and Acute Care Surgery, North Memorial Health Hospital, Robbinsdale, MN USA

**Keywords:** Anticoagulants, Hemostasis, Hemorrhage, Warfarin, Blood coagulation factors

## Abstract

**Background:**

Prothrombin Complex Concentrates (PCC) are prescribed for emergent warfarin reversal (EWR). The comparative effectiveness and safety among PCC products are not fully understood.

**Methods:**

Patients in an academic level one trauma center who received PCC3 or PCC4 for EWR were identified. Patient characteristics, PCC dose and time of dose, pre- and post-INR and time of measurement, fresh frozen plasma and vitamin K doses, and patient outcomes were collected. Patients whose pre-PCC International Normalized Ratio (INR) was > 6 h before PCC dose or the pre-post PCC INR was > 12 h were excluded. The primary outcome was achieving an INR ≤ 1.5 post PCC. Secondary outcomes were the change in INR over time, post PCC INR, thromboembolic events (TE), and death during hospital stay. Logistic regression modelled the primary outcome with and without a propensity score adjustment accounting for age, sex, actual body weight, dose, initial INR value, and time between INR measurements. Data are reported as median (IQR) or n (%) with *p* < 0.05 considered significant.

**Results:**

Eighty patients were included (PCC3 = 57, PCC4 = 23). More PCC4 patients achieved goal INR (87.0% vs. 31.6%, odds ratio (OR) = 14.4, 95% CI: 3.80–54.93, *p* < 0.001). This result remained true after adjusting for possible confounders (AOR = 10.7, 95% CI: 2.17–51.24, *p* < 0.001). The post-PCC INR was lower in the PCC4 group (1.3 (1.3–1.5) vs. 1.7 (1.5–2.0)). The INR change was greater for PCC4 (2.3 (1.3–3.3) vs. 1.1 (0.6–2.0), *p* = 0.003). Death during hospital stay (*p* = 0.52) and TE (*p* = 1.00) were not significantly different.

**Conclusions:**

PCC4 was associated with a higher achievement of goal INR than PCC3. This relationship was observed in the unadjusted and propensity score adjusted results.

## Background

Critical bleeding events in patients receiving warfarin anticoagulation poses significant morbidity and mortality risk [1]. The annual incidence of major bleeding associated with warfarin reported in a pooled analysis of three clinical trials comparing warfarin to novel oral anticoagulants (NOACs) in atrial fibrillation patients was 3.09–3.57%, with intracerebral hemorrhage (ICH) and gastrointestinal (GI) bleeding having a reported incidence of 0.70–0.80% and 0.86–1.02%, respectively [2]. When critical bleeding occurs, rapid reversal of the anticoagulant effects of warfarin can slow or arrest progression of the bleed and allow for treatment interventions to correct the cause and source of the bleed.

The International Normalized Ratio (INR) is the currently recommended laboratory assessment used to determine reversal of warfarin anticoagulation [1]. Traditional strategies for reversal of warfarin’s anticoagulation effects and the lowering of INR include discontinuation of warfarin, administration of vitamin K to promote hepatic biosynthesis of vitamin K-dependent clotting factors II, VII, IX, and X [3], and fresh frozen plasma (FFP) to replace functional clotting factors [4]. These strategies, while effective, are limited because the time to reverse the INR is delayed over several hours [5]. The onset of action of intravenous (IV) vitamin K to lower the INR is one to 2 h, and longer when given orally (PO) [3, 6]. Fresh frozen plasma requires time for thawing and blood group matching and is administered in large volumes, usually over 30 min [4]. Risks associated with administration of FFP, including transfusion reactions, transmission of infections, transfusion related lung injury, and the risk of complications associated with volume overload secondary to the large fluid volume of FFP [4].

Recent strategies to rapidly reverse warfarin anticoagulation include the administration of prothrombin complex concentrate (PCC), also known as factor IX complex, in the setting of critical bleeding or emergent surgery. These products can be administered more quickly and normalize the INR more rapidly than FFP [7]. Prothrombin complex concentrate products are derived from pooled human plasma and are free of viral contaminants. They vary in composition and amount of clotting factors [1]. All PCC products contain factors II, IX, and X, but 3-factor PCC (PCC3) products differ in that they contain low amounts of factor VII. Four factor PCC products contain higher concentrations of factor VII [7]. Several recent guidelines recommend PCC4 (along with vitamin K), as a first line agent for emergent warfarin reversal (EWR). Plasma is recommended as an alternative if PCC4 is not available [1, 4, 8–13]. Prior to 2013, only PCC3 products were available in the United States (US). These products were used off-label and with no guidance for dosing for EWR. In April 2013, the US Food and Drug Administration (FDA) approved a PCC4 product for warfarin reversal that contains clotting factors II, VII, IX, and X, as well as the anticoagulant proteins C and S, heparin, antithrombin III, and human albumin [14]. This product was approved based on randomized, noninferiority, plasma-controlled studies that found more rapid reversal of the INR with PCC4 than plasma [15, 16].

The aim of this study is to describe and characterize the differences in effectiveness of INR lowering between PCC4 and PCC3 in patients who required EWR. Given the compositional differences in the PCC products, it is important to know if there are differences in clinical response for effective and safe use of these products. Our primary outcome is achieving an INR of 1.5 or less after the first dose of PCC. Additionally, we explored the change in INR, thromboembolic events (TE), death during hospital stay and length of stay between the PCC3 and PCC4 groups. To maximize the comparative effectiveness of the response of PCC on INR while minimizing effects of external factors, we restricted our patient inclusion to those who received PCC within 6 h of the baseline INR and the pre- and post-PCC INR measurement to be no greater than 12 h.

## Methods

### Study population

This research was conducted at North Memorial Medical Center, an American College of Surgeons verified Level 1 Trauma Center. Institutional Review Board (IRB) for both North Memorial and the University of Minnesota found this research to be exempt from review. Deidentified patient data were collected from an electronic medical record (EMR) database during retrospective chart review. Patients who received PCC3 or PCC4 between August 29, 2007 and June 30, 2014 were reviewed for inclusion and exclusion criteria. Patients were included who had documented warfarin usage prior to admission, required EWR, an initial INR ≥ 1.6, received either PCC3 or PCC4 at a dose range of 20–50 units/kg with an allowance for rounding to the nearest 500 unit vial, at least one INR value obtained pre PCC administration, and at least one INR obtained post PCC administration. Kcentra®, the PCC4 product used, is dosed in factor IX units, and contains 200 to 500 units of factor VII per 500 unit vial [14]. Profilnine®, the PCC3 product used, contains no more than 175 factor VII units per 500 factor IX units [17]. Patients were excluded if they had an INR ≤ 1.5 before PCC administration, received recombinant activated factor VII (rFVIIa), did not have an INR measurement before or after PCC administration, pre-PCC dose INR was drawn greater than 6 h from the dose given, or greater than 12 h elapsed from the pre-PCC dose INR to post-PCC dose INR. Administration of FFP units and vitamin K dose and route were not standardized by treatment protocol and were left to the discretion of the provider.

The following patient data were retrieved from the EMR: 1) Demographic: age, sex, weight, warfarin indication, and bleed type; 2) Coagulation parameters: INR pre and post administration of either PCC3 or PCC4, and INR measurement times; 3) Reversal agent administration time and dose: PCC3, PCC4, FFP, vitamin K; 4) Patient outcome: death during hospital stay, hospital length of stay, and TE type and incidence. Several variables were derived from the EMR data, including change in INR, achievement of an INR ≤ 1.5, and PCC dose per kilogram of actual body weight. Bleed types were categorized into three groups: ICH, GI, and other bleeding. The laboratory reported INR value was capped at 8; the consideration for this is detailed in the statistical methods section. INR monitoring was done without a systematic schedule after reversal agent administration. The treating physicians diagnosed TE upon clinical examination rather than systematic screening driven by hospital protocol. Patients missing data pertaining to the inclusion criteria were excluded.

### Statistical methods

Descriptive statistics are reported as median and interquartile range for continuous data. Categorical data are reported as number and percent. Clinically relevant differences between groups are assessed highlighting the differences in medians or proportions.

Because these data reflect real-world clinical care, and the patients were not randomized into treatment groups, patients given one PCC treatment may differ systematically from the patients given the other [18]. To account for this and to make direct comparisons between PCC groups more meaningful, a propensity score was estimated by regressing the PCC treatment on patients’ observed covariates using logistic regression. The propensity score accounted for age, sex, actual body weight, PCC dose, initial INR value, and time from the first and second INR measurement. The propensity score estimate was then used in regression models as a covariate after checking the comparability of distribution of scores between the groups.

Our primary goal was to compare the odds of achieving the goal INR ≤ 1.5 after administration of PCC3 or PCC4. An unadjusted odds ratio (OR) and propensity-score adjusted odds ratio (AOR) were estimated via logistic regression. Univariate logistic regression examined the associations between achieving goal INR and sex, age, weight, PCC dose, PCC type, bleed type, warfarin indication, the units of FFP given, pre-PCC dose INR value, and time elapsed from the pre-PCC dose INR to post-PCC dose INR. We included a maximum 12-h window from pre-PCC to post-PCC INR measurement. Goal INR achievement between the groups was stratified by two and three-hour blocks of time from the dose to the post-PCC INR measurement time to investigate whether differences in follow-up INR measurement times could affect overall goal attainment in each PCC group. To account for confounding we stratified our results by bleed type and included it in models.

Continuous secondary outcomes such as change in INR and post-PCC INR measurements were modeled using linear regression with and without propensity score adjustment using those given PCC3 as the reference group. Other secondary outcomes such as TE, death during hospital stay, and length of stay were reported as counts. It is important to note that the linear models analyze mean values rather than medians as was reported in the descriptive results.

The impact of Vitamin K was considered in the models. Models were examined with and without INR values of 8 or greater. The censored INR values were replaced with imputed INR values ranging from 10 to 15 to estimate the effect censoring had on the results. Interactions between the predictor variables were also considered. Goodness of fit tests and regression model diagnostics were performed to ensure the appropriateness of the models [19, 20]. The a priori significance level was set to 0.05. Data were analyzed using R version 3.5.3 [21].

## Results

**Fig. 1:**
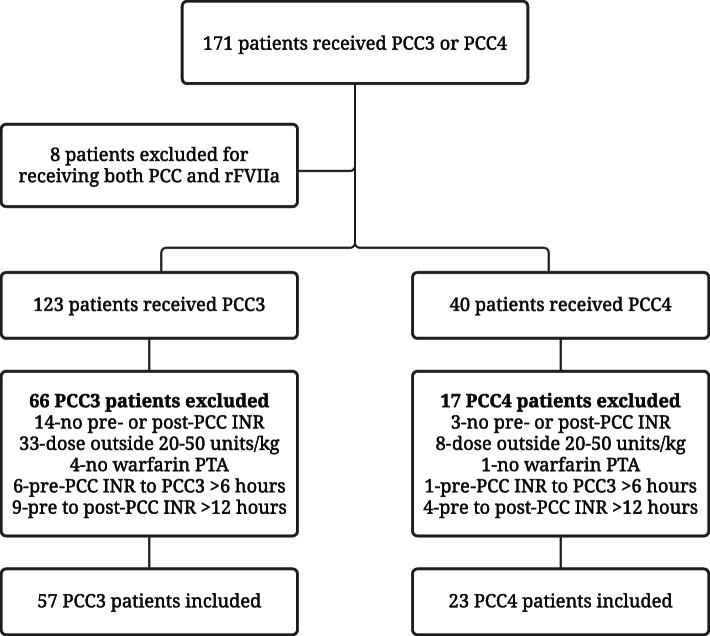
Flow diagram showing patient selection. INR = International Normalized Ratio, PCC3 = 3 factor prothrombin complex concentrate, PCC4 = 4 factor prothrombin complex concentrate, PTA = prior to admission, rFVIIa = recombinant factor VII (activated)

Patients who received either PCC3 or PCC4 (*n* = 171) were identified after chart review. Eighty patients were included in the final analysis: 57 received PCC3 and 23 received PCC4 (Fig. 1). There are some key differences between PCC groups to highlight. The median age of the PCC3 group was 8 years older than the PCC4 group. There were fewer females in the PCC3 group (36.8%) than the PCC4 group (47.8%). The median weight was slightly higher in the PCC3 group (Table 1).

**Table 1:** Demographics and Indications

Variables	PCC3 (***n*** = 57)	PCC4 (***n*** = 23)
**Characteristics**		
Age (years)^a^	74.0 (62.0–80.0)	66.0 (57.0–82.0)
Weight (kg)^a^	81.4 (72.1–94.4)	77.8 (64.7–97.8)
Sex, n (%)		
Male	36 (63.2%)	12 (52.2%)
Female	21 (36.8%)	11 (47.8%)
**Indication for warfarin, n (%)**		
Atrial Arrhythmias	32 (56.1%)	8 (34.8%)
DVT/PE^b^	7 (12.8%)	4 (17.4%)
Valve replacement	6 (10.5%)	5 (21.7%)
Ischemia CVA	4 (7.0%)	1 (4.3%)
Other	8 (14.0%)	5 (21.7%)
**Bleed type, n (%)**		
Intracranial	31 (54.4%)	19 (82.6%)
Gastrointestinal	10 (17.5%)	2 (8.7%)
Other	16 (28.1%)	2 (8.7%)

*CVA* cerebrovascular accident, *DVT/PE* deep vein thrombosis and/or pulmonary embolism, *EWR* emergent warfarin reversal, *PCC3* 3-factor prothrombin complex concentrate, *PCC4* 4-factor prothrombin complex concentrate

^a^Age and Weight given as median (25th–75th percentiles)

^b^Prophylaxis or treatment

Table 2 lists the anticoagulation parameters and reversal agents administered. The median dose for PCC was higher in the PCC4 group in term of units (PCC3: 2000 (1530–2500) vs. PCC4: 2595 (1880–3307)), and units/kg (PCC3: 21.5 (20.4–25.9) vs. PCC4: 29.3 (25.9–37.3)), and units/kg to INR (PCC3: 7.9 (5.6–10.5) vs. PCC4: 8.2 (7.0–10.2)), where units/kg to INR reflects the dosing of PCC4 as weight based and stratified by initial INR. The route and amount of vitamin K varied between groups. Fewer patients were administered IV vitamin K than the PCC4 group. More subcutaneous, and oral doses were administered to the PCC3 group of vitamin K, and more patients received no vitamin K at all compared the PCC4 group. Also, the median dose of vitamin K in the PCC3 group (5 mg) was half that given to the PCC4 group (10 mg). A higher proportion of patients in the PCC3 group (59.6%) were administered FFP than the PCC4 group (30.4%) during hospital stay, and between the first and second INR (PCC3: 26.3% vs. PCC4: 8.7%). Similar proportions of patients received packed red blood cell (RBC) transfusions in each group during their hospital stay (PCC3: 36.8% vs. PCC4: 30.4%).

**Table 2:** Anticoagulation parameters and reversal agents administered

Agents	PCC3 (***n*** = 57)	PCC4 (***n*** = 23)
**PCC dose**		
Dose (units)	2000 (1530–2500)	2595 (1880–3307)
Dose by weight (units/kg)	21.5 (20.4–25.9)	29.3 (25.9–37.3)
Dose by pre-PCC INR (units/INR)	645.2 (438.3–982.8)	674.3 (576.5–870.0)
Dose by weight/pre-PCC INR (units/kg/INR)	7.9 (5.6–10.5)	8.2 (7.0–10.2)
**INR**		
INR prior to reversal	2.8 (2.1–4.1)	3.7 (2.6–4.9)
INR post first dose	1.7 (1.5–2.0)	1.3 (1.3–1.4)
INR change pre to post	1.1 (0.6–2.0)	2.3 (1.2–3.3)
**Time (minutes)**		
Minutes from pre-PCC INR to dose given	78 (56.0–113.0)	73 (40.0–108.5)
Minutes from dose given to post-PCC INR (6 h or less)	93 (46.0–228.0)	226 (156.5–368.5)
Minutes from pre- to post-PCC INR (12 h or less)	215 (133.0–326.0)	296 (241.0–483.0)
**Vitamin K routes, n (%)**		
Intravenous	36 (63.2%)	21 (91.3%)
Oral	9 (15.8%)	1 (4.3%)
Subcutaneous	4 (7.0%)	0 (0.0%)
None	8 (14.0%)	1 (4.3%)
**Vitamin K given during stay (mg)**	5.0 (2.0–10.0)	10.0 (7.5–10.0)
**FFP given during stay (yes/no), n (%)**	34 (59.6%)	7 (30.4%)
Units given in those receiving FFP (range)	(1–12)	(1–6)
**FFP given between INR 1 and 2 (yes/no), n (%)**	15 (26.3%)	2 (8.7%)
Units given in those receiving FFP (range)	(1–4)	(1, 2)
**RBC given during stay (yes/no), n (%)**	21 (36.8%)	7 (30.4%)
Units given in those receiving RBC (range)	(1–12)	(1–4)

Median (25th–75th percentiles) given unless specified, *FFP* fresh frozen plasma, *INR* international normalized ratio, *PCC* prothrombin complex concentrate, *PCC3* 3-factor PCC, *PCC4* 4-factor PCC, *RBC* red blood cells

**Fig. 2:**
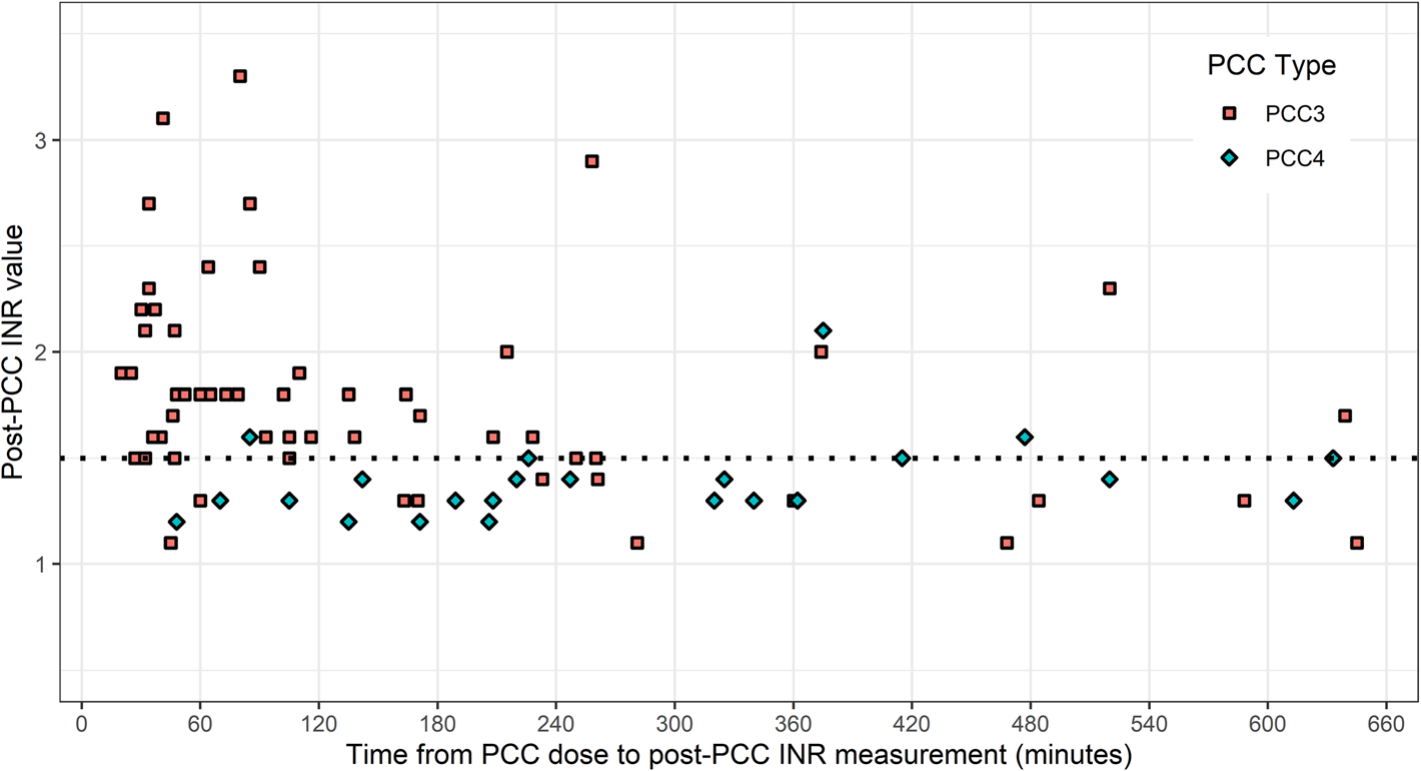
Post-PCC dose INR measurements over time. The dotted line represents the hospital- specific goal (INR ≤ 1.5). Note: One patient given PCC3 whose post-PCC INR measurement was above the clinical laboratory maximum reported value of 8 is omitted from the figure. Although we included a 12-h time window, no patients had a follow-up INR measurement after 11 h. INR = International Normalized Ratio, PCC = prothrombin complex concentrate, PCC3 = 3 factor PCC, PCC4 = 4 factor PCC

The median pre-PCC dose INR was lower in the PCC3 group, 2.8 (2.1–4.1), than the PCC4 group 3.7 (2.6–4.9). However, after the dose, the INR after the first PCC dose was higher in the PCC3 group, 1.7 (1.5–2.0), than the PCC4 patients, 1.3 (1.3–1.4). This resulted in a lower median change in INR in the PCC3 group, 1.1 (0.6–2.0), than patients given PCC4, 2.3 (1.2–3.3). The post-PCC dose INR measurements over time in minutes post-PCC dose are displayed in Fig. 2. Both groups were given PCC in a similar time (minutes) once the initial INR was measured (PCC3: 78 (56.0–113.0) vs. PCC4: 73 (40.0–108.5)). The time from the dose to the next INR measurement differed widely (PCC3: 93 (46.0–228.0) vs. PCC4: 226 (156.5–368.5)), as well as the time from the first and second INR measurements (PCC3: 215 (133.0–326.0), PCC4: 296 (241.0–483.0)).

### Clinical outcomes

Patient outcomes are presented in Table 3. A greater proportion of patients who received PCC4 achieved goal INR (PCC3: 18 (31.6%) vs. PCC4: 20 (87%), *p* < 0.001). Univariate regression found the unadjusted odds ratio of achieving an INR ≤ 1.5 after the first PCC dose is 14 times higher in those receiving PCC4 than PCC3 (OR = 14.4, 95% CI: 3.80–54.93, *p* < 0.001). Attainment of goal INR also favored PCC4 when time from dose to post-PCC INR measurement was stratified by two and three-hour blocks. Univariate analysis also found that females appeared to be more likely to achieve an INR ≤ 1.5 after the first dose compared to males (OR = 3.48, 95% CI: 1.36–8.90, *p* = 0.009). Time from pre- to post-PCC INR was also significantly associated with achieving target INR. As expected, every hour that passed from pre- to post-PCC INR increased the odds of achieving an INR ≤ 1.5 by 28% (OR = 1.28, 95% CI: 1.08–1.53, *p* = 0.005). After propensity score adjustment for age, sex, actual body weight, dose, initial INR value, and time from the first and second INR measurement patients given PCC4 were still much more likely to reach goal INR (AOR = 10.7, 95% CI: 2.17–51.24, *p* < 0.001).

**Table 3:** Patient Outcomes

Outcome	PCC3 (***n*** = 57)	PCC4 (***n*** = 23)	***p***
**Patients achieving INR ≤ 1.5, n (%)**	18 (31.6%)	20 (87.0%)	< 0.001
Intracranial bleeding	10 (32.3%)	16 (84.2%)	
Gastrointestinal bleeding	4 (40.0%)	2 (100%)	
Other bleeding	4 (75%)	2 (100%)	
**Death during hospitalization, n (%)**	14 (24.7%)	8 (34.8%)	0.52
Intracranial bleeding	10 (32.3%)	7 (36.8%)	
Gastrointestinal bleeding	1 (10.0%)	0 (0%)	
Other bleeding	3 (75%)	1 (50%)	
**Length of stay**			
Overall	6.0 (4.0–11.0)	6.0 (2.5–10.0)	0.47
Survivors	6.5 (4.0–10.0)	6.0 (3.0–9.5)	0.52
**Thromboembolic events, n (%)**	5 (8.8%)	2 (8.7%)	1.0

*INR* international normalized ratio, *PCC* prothrombin complex concentrate, *PCC3* 3-factor PCC, *PCC4* 4-factor PCC

Linear regression found the mean change in INR of the reference group, those given PCC3, was 1.64. The mean change in the PCC4 group was 0.91 higher on average than the PCC3 group (*p* = 0.03). After propensity score adjustment, the estimated mean change in INR in the PCC3 group was 1.39, and the PCC4 group was 0.30 higher on average (*p* = 0.56). The post-PCC INR in the PCC3 group was 1.90. This was 0.51 lower on average in the PCC4 group (*p* = 0.01). After propensity score adjustment, the post-PCC INR in the PCC3 group was 1.98, and the PCC4 group was 0.45 lower on average (*p* = 0.21). A summary of the regression models are presented in Table 4.

**Table 4:** Regression Models

Factors	OR (95% CI)	***P***
**Univariate Logistic Regression for INR Goal Met**		
PCC4	14.44 (3.80–54.93)	< 0.001
Female	3.48 (1.36–8.90)	0.009
Age	1.00 (0.98–1.03)	0.85
Weight (kg)	1.00 (0.98–1.02)	0.71
Dose (per 500 unit vial)	1.23 (0.92–1.63)	0.16
Bleed type		
Other	*Ref.*	
Intracranial	2.17 (0.70–6.68)	0.18
Gastrointestinal	2.00 (0.45–8.94)	0.36
Initial INR	0.78 (0.61–1.01)	0.06
FFP units used	1.05 (0.67–1.63)	0.84
Time from pre- to post-PCC INR (in hours)	1.28 (1.08–1.53)	0.005
Survivors	1.12 (0.42–3.00)	0.82
Length of stay, all	0.95 (0.88–1.03)	0.22
Length of stay, survivors	0.94 (0.85–1.05)	0.27
Thromboembolism, yes	0.43 (0.10–1.79)	0.25
**PS Adjusted Logistic Regression**	**AOR (95% CI)**	
PCC4	10.55 (2.17–51.24)	< 0.001
**Linear Regression**	**Coefficient (95% CI)**	
Intercept (mean INR change PCC3)	1.64 (1.22–2.07)	< 0.001
mean INR change PCC4	0.91 (0.12–1.70)	0.03
Intercept (post-PCC INR PCC3)	1.90 (1.69–2.12)	< 0.001
post-PCC INR PCC4	−0.51 (−0.91- -0.12)	0.01
**PS Adjusted Linear Regression**	**Coefficient (95% CI)**	
Intercept (mean INR change PCC3)	1.39 (0.89–1.89)	< 0.001
mean INR change PCC4	0.30 (−0.74–1.33)	0.56
Intercept (post-PCC INR PCC3)	1.98 (1.73–2.23)	< 0.001
post-PCC INR PCC4	−0.45 (− 0.85- -0.19)	0.21

*AOR* adjusted odds ratio, *CI* confidence interval, *INR* international normalized ratio, *FFP* fresh frozen plasma, *NA* not applicable, *OR* odds ratio, *PCC* prothrombin complex concentrate, *PCC3* 3-factor PCC, *PCC4* 4-factor PCC, *PS* propensity score, *Ref.* reference group

We observed death during hospital stay in 10% more patients given PCC4 (PCC3: 24.7% vs. PCC4: 34.8%, *p* = 0.52). Length of stay did not differ meaningfully between groups for all patients or survivors only. Thromboembolic events were similar between groups with five (8.8%) in the PCC3 group compared to two (8.7%) in the PCC4 group (*p* = 1.0).

## Discussion

In the setting of critical bleeding associated with warfarin anticoagulation, rapid reversal of the effects of warfarin and correction of the INR are desired with the goal of slowing the progression of the bleed, achieving hemostasis, and allowing surgical intervention when needed. Several coagulation factor products are available and have been used to provide rapid reversal of warfarin anticoagulation. These products have varying composition of coagulation factor components. There is a need for better understanding of the similarities and differences in these agents in terms of anticoagulation reversal response and real-world effectiveness and safety outcomes.

Recent guidelines recommend the use of a PCC4 product, plus vitamin K, for urgent reversal of the effects of warfarin with INR as a measure of effective reversal [1, 4, 8–13]. The recommendation for a PCC4 product over PCC3 is based on the theoretical advantage of the additional factor VII component and randomized controlled trials (RCT) that compared PCC4 to FFP [15, 16], and was not based on any prospective study comparing EWR with PCC3 vs. PCC4 products. In the US, PCC4 has surpassed PCC3 for EWR with FFP as a second line agent. A recent survey of 281 critical care and emergency medicine pharmacists found, 92.9% reported use of PCC4 for warfarin reversal. Interestingly, only 58.7% reported using the labeled weight-based dosing strategy; of those not following label, 30.6% used a fixed-dose regimen, commonly a single dose of 1500 units [22].

This research evaluating the effectiveness and safety of PCC3 or PCC4 for EWR found patients treated with PCC4 were 14 times more likely to achieve a goal INR ≤ 1.5 after the first PCC dose compared to those given PCC3. The proportion of patients who reached the goal INR was higher with PCC4 (87% vs. 31.6%, *p* < 0.001). The differences in mean change in INR, and post-PCC INR lost statistical significance after propensity score adjustment. While this is not a RCT designed and powered to detect these differences, this indicates the adjunct treatments and demographic differences included in the propensity score influence the INR reduction in patients given PCC products. Also, there is likely suboptimal statistical power given the small sample size to detect statistically significant differences once adjustments have been made to the analysis. Statistically significant and clinically relevant differences from the univariate regression indicate the need to account for sex and time from pre- to post-PCC INR in future analysis regardless of study type.

The primary outcome of this research was meeting INR goal, which reflects the outcome used in clinical practice. Ideally, a RCT with a standardized treatment protocol accounting for PCC timing and adjunct treatments would be needed to confirm the differences in INR reduction between the PCC products. However, given the critical need to reverse the effects of warfarin in the setting of an emergent bleeding episode, a randomized trial would be difficult to implement.

Several previous studies compare the effectiveness of PCC3 and PCC4 for EWR with the outcome of INR lowering response and/or clinical outcomes [23–29]. The inclusion criteria for these studies vary, but all were in adult patients needing reversal of warfarin for bleeding, surgical intervention, and/or trauma associated bleeding. These studies did not control for pre-defined times between dose and INR measurements as ours did, thus allowing for varied durations of time for coagulation reversal from PCC and other reversal agents. The only systematic review and meta-analysis evaluating PCC3 vs. PCC4 compares separate studies from various institutions rather than head-to-head comparisons [30]. Our study is similar to these other studies regarding retrospective design, PCC products compared, and the adult population. However, our comparison differed from those of others (discussed below) who have reported similar comparisons in that our inclusion criteria was more strict in that the time from pre-PCC INR to post-PCC INR was limited to 12 h, the PCC dose administration in relation to the pre-PCC INR was limited to 6 h. Further, we employed regression modeling and propensity score adjustment not commonly employed in other published comparisons.

The findings from other published studies comparing the effects of PCC3 and PCC4 on warfarin anticoagulation reversal based on INR response were mixed. Al-Majzoub, et al. in a single-center study in patients actively bleeding on warfarin found the proportion of patients achieving a goal INR ≤ 1.3 was greater in those who received PCC4 than PCC3 (PCC4: 15/18 (83.3%) vs. PCC3 15/35 (42.9%), *p* < 0.01) [25]. Mangram, et al. studied trauma patients from two centers on oral anticoagulants (warfarin or rivaroxaban). Excluding those on rivaroxaban, successful reversal (INR of < 1.5) for the patients on warfarin occurred more frequently in those given PCC4 compared to PCC3 (PCC4: 13/16 (81%) vs. PCC3: 23/45 (51%), *p* = 0.043) [26]. Holt, et al. studied multiple centers with warfarin reversal defined as an INR ≤ 1.3. Regardless, a greater proportion of PCC4 patients achieved goal INR compared to PCC3 patients (PCC4: 48/57 (84.2%) vs. PCC3:40/77 (51.9%), *p* < 0.001) (patient counts calculated from percentages and total patient number) [27]. DeAngelo, et al. reported analysis from two centers with institutions with the same formulary and protocols. Adequate INR reversal (INR of ≤1.5) was more common with PCC4 than PCC3 (PCC4: 28/32 (87.5%) vs. PCC3: 26/57 (45.6%), *p* < 0.001) (patient counts calculated from percentages and total patient number) [28]. Voils, et al., in a single-center study, reported similar frequencies of patients achieving a 30 min post-PCC INR of ≤1.5 (PCC4: 47/56 (84%) vs. PCC3: 87/109 (80%), *p* = 0.52) [23]. Jones, et al. published a multicenter, propensity-matched study with a primary outcome of INR ≤ 1.4 and found similar proportions achieved goal INR in the unmatched analysis (PCC4: 58/64 (90.6%) vs. PCC3: 72/84 (85.7%), *p* = 0.37) and matched analysis (PCC4: 35/38 (92.1%) vs. PCC3: 32/38 (84.2%), *p* = 0.48) [24].

Limitations to our research include a single-center design, so we cannot be certain of the generalizability of our findings to populations outside of ours. Causal relationships between PCC type and outcomes are difficult to establish due to the retrospective nature of the study. Temporal differences between groups could account for differences in INR response between PCC3 and PCC4 in our study. Off label PCC3 was used for warfarin reversal at our site before it was replaced with a PCC4 product approved for warfarin reversal in the US. Therefore, there were no labeled dosing recommendations for PCC3 unlike PCC4. Additionally, the differences in the PCC groups in demographic and INR-related results could bias the outcomes. We accounted for this as best we could by using propensity score adjustments. The propensity scores allowed us to compare PCC groups more directly, but they can only adjust for observed covariates, and propensity score methods work better in larger samples for distributional balance. We chose not to match patients based on their propensity score because we may have left some patients out of the analysis. The clinical INR measurements did not allow us to investigate its decrease over time, which may not be linear. Additionally, the clinical goal was to achieve an INR ≤ 1.5 regardless of the decrease in INR, so the effect of PCC on the INR value is difficult to establish.

An additional limitation of the data was the lack of a standardized assessment of effectiveness of major bleeding management in clinical practice to use hemostatic efficacy as a study outcome. Hemostatic efficacy is a benchmark outcome for trials adopted by The International Society on Thrombosis and Haemostasis (ISTH) for new reversal agents [31]. However, specific bleeding outcomes were not documented during clinical care aside from RBC transfusions, and therefore this analysis could not assess the endpoints listed by the ISTH.

While the INR has been and continues to be the standard measure for assessing PCC use for the correction of coagulopathy in the setting of critical bleeding [1, 4], a recent review of the literature evaluating the clinical utility of INR to guide PCC use in the reversal of warfarin found limited evidence to support this practice [32]. Alternative measures that have been considered for assessing warfarin reversal include global coagulation assays such as thrombin generation assay and thromboelastography [33–35]. An in vitro model of warfarin reversal compared one PCC3 and two PCC4 products at concentrations of 0.5, 1.0, and 1.5 U/ml to assess INR response and thrombin generation (reported as endogenous thrombin potential) and thromboelastometry (reported as clotting time). While there was a dose dependent response to INR lowering effect to reversing warfarin, endogenous thrombin potential and clotting time were equally corrected at the lowest concentration [36].

This study, like others and as recommended by the guidelines, assessed the change in INR as the outcome measure for effectiveness. While the results found PCC4 was more effective at achieving our defined endpoint of an INR of less than or equal to 1.5, whether this translates to unequal effectiveness in correction of warfarin anticoagulation between PCC3 and PCC4 in not known. Further research evaluating how INR changes and global coagulation assay changes in response to factor products is needed for to evaluate dosing of these products and protect our patients from overcorrection of anticoagulation and the risk of thromboembolic events.

## Conclusions

PCC4 was associated with a higher achievement of goal INR than PCC3. This relationship was observed in the unadjusted and propensity score adjusted results. However, the mechanism for this finding is not fully known. Compositional differences between the PCC products could be the cause, but it is not possible to establish the causal relationship with a single-center, retrospective study.

## Data Availability

The datasets during and/or analyzed during the current study available from the corresponding author on reasonable request.

## Abbreviations

AOR: Adjusted odds ratio
EMR: Electronic medical record
EWR: Emergent warfarin reversal
FDA: Food and Drug Administration
FFP: Fresh frozen plasma
GI: Gastrointestinal
ICH: Intracerebral hemorrhage
INR: International normalized ratio
IRB: Institutional review board
IV: Intravenous
NOACs: Novel oral anticoagulants
OR: Odds ratio
PCC: Prothrombin complex concentrate
PCC3: Three-factor prothrombin complex concentrate
PCC4: Four-factor prothrombin complex concentrate
rFVIIa: Recombinant activated factor VII
TE: Thromboembolic events
US: United States
